# Impact of home and community-based services on hospitalisation and institutionalisation among individuals eligible for long-term care insurance in Japan

**DOI:** 10.1186/1472-6963-10-345

**Published:** 2010-12-22

**Authors:** Naoki Tomita, Kimio Yoshimura, Naoki Ikegami

**Affiliations:** 1 Department of Health Policy & Management, Keio University School of Medicine, Tokyo, JAPAN

## Abstract

**Background:**

This population-based retrospective cohort study aimed to clarify the impact of home and community-based services on the hospitalisation and institutionalisation of individuals certified as eligible for long-term care insurance (LTCI) benefits.

**Methods:**

Health insurance data and LTCI data were combined into a database of 1,020 individuals in two farming communities in Hokkaido who were enrolled in Citizen's Health Insurance. They had not received long-term care services prior to April 1, 2000 and were newly certified as eligible for Long-Term Care Insurance benefits between April 1, 2000 and February 29, 2008. The analysis covered 565 subjects who had not been hospitalised or institutionalised at the time of first certification of LTCI benefits. The adjusted hazard ratios (HRs) of hospitalisation or institutionalisation or death after the initial certification were calculated using the Cox proportional hazard model. The predictors were age, sex, eligibility level, area of residence, income, year of initial certification and average monthly outpatient medical expenditures, in addition to average monthly total home and community-based services expenditures (analysis 1), the use or no use of each type of service (analysis 2), and average monthly expenditures for home-visit and day-care types of services, the use or no use of respite care, and the use or no use of rental services for assistive devices (analysis 3).

**Results:**

Users of home and community-based services were less likely than non-users to be hospitalised or institutionalised. Among the types of services, users of respite care (HR: 0.71, 95% confidence interval [CI]: 0.55-0.93) and rental services for assistive devices (HR: 0.70, 95% CI: 0.54-0.92) were less likely to be hospitalised or institutionalised than non-users. For those with relatively light needs, users of day care were also less likely to be hospitalised or institutionalized than non-users (HR: 0.77, 95% CI: 0.61-0.98).

**Conclusions:**

Respite care, rental services for assistive devices and day care are effective in preventing hospitalisation and institutionalisation. Our results suggest that home and community-based services contribute to the goal of the LTCI system of encouraging individuals certified as needing long-term care to live independently at home for as long as possible.

## Background

The social costs of hospitalisation and institutionalisation are growing [1-3], and the majority of elderly people would prefer to stay in their own homes, even if they have a serious disability [4]. Extending the period in which elderly people are able to live at home has thus become a very important issue. Japan's public long-term care insurance (LTCI) system was introduced in April 2000 from this perspective. LTCI, by making it easier for individuals certified as needing long-term care to use home and community-based services, aims to prevent decline of functional level and allow elderly people to live independently in their homes for as long as possible [5]. Hospitalisation or institutionalisation would therefore be an adverse event. Although hospitalisation is for providing medical services and institutionalisation is for providing long-term care services, for frail elderly it is difficult to distinguish between the two. This is especially the case in Japan, where admissions to hospitals are frequently made for social reasons--no family member to provide care or long waiting lists for nursing homes [6-8].

Hospitalisation and institutionalisation have been used as outcome measures to observe the impact of preventive interventions such as education, counselling, and assessment in various countries [9-17], but studies on the effects of home and community-based services have been limited. In Japan, the preventive effect of day care on institutionalisation was reported before the introduction of LTCI [18,19]. Following the introduction of LTCI, outcomes have focused not on admissions, but on changes in eligibility levels [20-24], moreover, these studies did not adjust either for the use of medical services or for medical condition. Reports in other countries include the effect of home-help for the elderly with dementia [25], and that of day care [26,27] or that of respite services combining day care and respite care [28], but these studies were confined to institutionalisation and did not include hospitalisation. On the other hand, Xu et al. [29] showed that a greater volume of attendant care, homemaking services and home-delivered meals was associated with a lower risk of hospitalisation. In this study, we decided to focus on the impact of home and community-based services on hospitalisation and institutionalisation after adjusting for the use of medical services by using health insurance data and LTCI data from a small community. Specifically, we selected as outcome admission to a hospital or a long-term care institution after being certified as eligible for LTCI benefits. Additional subgroup analyses were made in order to confirm whether home and community-based services are beneficial for older adults with only light care needs as suggested by previous studies [20,22,24]. Our sample was limited to a small community because national databases do not exist, and linkage of health insurance and LTCI data was only possible in the area studied.

## Methods

### Public long-term care insurance system

Japan's LTCI is compulsory for all citizens ≥ 40 years of age, and those who are eligible for its benefits are individuals aged ≥ 65 years who require long-term care as well as individuals aged 40-65 years who require long-term care for diseases related to ageing. It is managed by municipal government. Certification of eligibility and determination of the level of benefits are based on a nationally standardised assessment process. When the LTCI system was introduced, six eligibility levels were established: "Support level", in which assistance is needed, and "Care levels 1, 2, 3, 4 and 5", in which care is needed, with 1 being the lightest and 5 being the heaviest. These levels are primarily determined by a computerised algorithm that is based on the responses to the questionnaire on current physical and mental status (74 items). This algorithm was derived from a statistical analysis of care time and subjects' clinical characteristics. The levels grouped by the algorithm do not correspond to clinical prototypes. The final decision is made by a local committee of specialists (physicians, social workers and so forth), who take into consideration a report from the attending physician and 12 quantitative aspects focused mainly on behaviour [30,31]. In April 2006, the LTCI system was revised to seven levels, with the Support level reset as "Support level 1" and the majority in "Care level 1" regrouped to "Support level 2" [31,32]. Certification of long-term care benefits is made without consideration of the willingness and ability of the individual's family or friends to provide care, or the individual's income. Benefits are provided not as cash but as home and community-based or institutional services, with recipients paying 10% of the care cost [33,34].

### Health insurance

Enrolling in health insurance is mandatory for all residents in Japan [30]. Employees, together with their dependents, are enrolled in employees' health insurance, while the self-employed, people living on pensions, and others are enrolled in Citizen's Health Insurance (CHI), which is managed by municipalities [35]. Therefore, the majority of elderly people are enrolled in CHI [35]. The proportion of the population aged 70 and above who are enrolled in CHI nationally was 82.3% in 2006 [36,37]. In the area of the present study the figure was 80.5%. Most people aged ≥ 70 years pay 10% of medical charges as copayment.

### Study area

This study was conducted in two farming communities (Towns A and B) located in central Hokkaido, which is in the north of Japan. This area is becoming increasingly depopulated and there is a lack of public transportation.

Table 1 provides basic data for the study area, Hokkaido, and all of Japan, for the years 2001 and 2007. It includes the population [38,39], the proportion of the aged 65 and above [38,39], the proportion of the population aged 70 and above who are enrolled in CHI (2006 only) [36,37], the average monthly expenditures per individual enrolled under the medical care for the elderly program [40,41], the proportion of the individuals certified as being eligible for LTCI benefits among individuals aged ≥ 65 years according to each eligibility level [42,43], and average monthly expenditures per recipient of specific care service [44-46].

**Table 1:** Characteristics of study site, Hokkaido and all of Japan

		2001			2007		
		**Study site**	**Hokkaido**	**Japan**	**Study site**	**Hokkaido**	**Japan**

Population*^1^		10,013	5,667,024	126,478,672	9,104	5,571,770	127,066,178

Proportion of the aged 65 and above (%)*^1^		28.0	19.0	18.3	33.0	23.0	21.6

**Proportion of the population aged 70 and above who ** are enrolled in Citizen' s Health Insurance (%)*^2^		-	-	-	80.5	-	82.3

	Outpatient medical expenditures	22,943	21,971	21,812	23,941	22,275	23,030
**Average monthly expenditures per individual ** **enrolled under the medical care for the ** elderly program (yen)*3	Inpatient medical expenditures	38,011	37,208	26,761	39,628	45,607	33,683
	Total medical expenditures	60,954	59,179	48,573	63,568	67,882	56,713

	Support level*^6^	2.7 (21.0)	1.9 (14.0)	1.7 (13.1)	3.4 (18.3)	2.1 (12.2)	2.0 (12.2)
	Care level 1*^7^	3.5 (26.4)	4.3 (32.0)	3.8 (29.3)	4.6 (24.8)	5.8 (34.0)	5.1 (30.8)
**Proportion of the individuals certified as ** **being eligible for LTCI benefits among **	Care level 2	2.4 (18.0)	2.5 (18.6)	2.4 (18.9)	3.0 (16.3)	2.9 (17.2)	2.9 (17.7)
**individuals aged ≥65 years according to** eacheligibility level (%) and its distribution(%)^*4^	Care level 3	1.6 (12.3)	1.5 (11.4)	1.7 (13.0)	2.6 (13.9)	2.4 (14.3)	2.6 (15.6)
	Care level 4	1.5 (11.7)	1.5 (11.4)	1.7 (13.0)	2.1 (11.4)	1.9 (11.1)	2.1 (12.7)
	Care level 5	1.4 (10.6)	1.7 (12.6)	1.6 (12.7)	2.8 (15.4)	1.9 (11.2)	1.8 (11.0)
	
	Total	13.1 (100)	13.3 (100)	12.9 (100)	18.4 (100)	17.0 (100)	16.5 (100)

	Total amount of home and community-based services	36,017	42,190	46,870	35,042	42,383	49,018
	Total amount of institutional services	295,525	360,799	347,202	266,367	270,556	269,441
	Total amount of long-term care services	144,532	123,423	109,802	103,139	98,776	90,554
	
	Home and community-based services						
	Home-help*^8^	31,490	45,164	53,396	40,300	40,805	48,866
	Home-visit bathing*^9^	0	45,376	48,715	44,962	52,359	56,356
	Visiting nurse*^9^	43,964	37,240	42,223	24,264	37,751	41,867
	Visiting rehabilitation*^9^	n/a	21,277	21,470	n/a	24,276	25,264
	Management & guidance*^9^	13,651	10,098	9,773	10,882	9,229	10,067
**Average monthly expenditures per recipient **	Day care*^10^	35,261	42,566	52,608	34,242	53,142	67,279
of specific care service (yen)*^5^	Day rehabilitation*^9^	39,469	57,469	68,512	36,577	58,584	67,341
	Respite care*^11^	106,800	93,959	83,162	80,232	82,583	87,963
	Rental services for assistive devices*^9^	12,359	13,461	14,349	12,202	12,163	14,858
	Small scale community-based multiple services centres*^9^	n/a	n/a	n/a	n/a	168,732	173,167
	
	Institutional services						
	Group homes for the elderly with dementia*^12^	0	239,865	238,750	253,656	261,843	262,342
	Residential care facilities for the elderly requiring care*^13^	189,171	157,917	185,640	146,515	153,129	176,692
	Nursing homes*^14^	273,710	324,612	329,049	262,478	253,480	260,221
	Health services facilities for the elderly	282,363	343,379	340,590	247,217	275,097	274,100
	Sanatorium-type medical care facilities	378,974	458,238	443,415	388,618	397,802	387,194

*1 Data from Japan Geographic Data Center, "Jyumin Kihon Daicho Jinko Yoran (Population summary of basic residents' register)" at the end of the each fiscal year.

*2 Proportion of the population aged 70 and above who are enrolled in Citizen's Health Insurance (%) = The population of the Citizen's Health Insurance member aged 70 and above / The total population of the persons aged 70 and above. Data at the end of the fiscal year 2006 is used.

*3 Data from All-Japan Federation of National Health Insurance Organizations, "Kokumin-Kenko-Hoken no Jittai (Situation of Citizen' s Health Insurance)". Data for study site, Hokkaido and Japan for 2002 is substituted for those for 2001. Data for "Sorachi Chubu Koiki Rengo", which includes study site, is substituted for the study site.

*4 Proportion of the individuals certified as being eligible for LTCI benefits among individuals aged ≥65 years (%) = the number of the all aged individuals certified as long-term care / the number of the insured individuals aged 65 and above. Data for Hokkaido and Japan from Japan Ministry of Health, Labour and Welfare, "Report on the Status of the Long-term Care Insurance Project" at the end of the each fiscal year.

*5 Data for study site for 2007 excludes March 2008. Data for Hokkaido and Japan from Japan Ministry of Health, Labour and Welfare, Ministry Secretariat, Statistics and Information Department, "Survey of Long-term Care Beneft Expenditures". Data for Hokkaido for 2002 is substituted for that for 2001.

*6 Support level for 2007 means Support level 1.

*7 Care level 1 for 2007 includes Support level 2.

*8 "Preventive care" and "Night care" are included for 2007.

*9 "Preventive care" is included for 2007.

*10 "Preventive care" and "Services for the elderly with dementia" are included for 2007.

*11 Consists of regular and medically-related Respite care. "Preventive care" is included for 2007.

*12 Counted as institutional services in this study. "Preventive care" is included for 2007.

*13 Counted as institutional services in this study. "Preventive care" and "Community-based residential care facilities for the elderly requiring care" are included for 2007.

*14 "Community-based welfare facilities for the elderly requiring care" are included for 2007.

All years are fiscal years; April to March.

Compared with the national average, the percentage of elderly people in this area was high. Outpatient medical expenditures per individual enrolled under the medical care for the elderly program were about the same level, but inpatient medical expenditures were higher. The proportion of Support level certification was higher, but the Care level was about the same as for the nation. The home and community-based and institutional services expenditures per recipient of long-term care were lower.

### Subjects (Figure 1)

**Figure 1:**
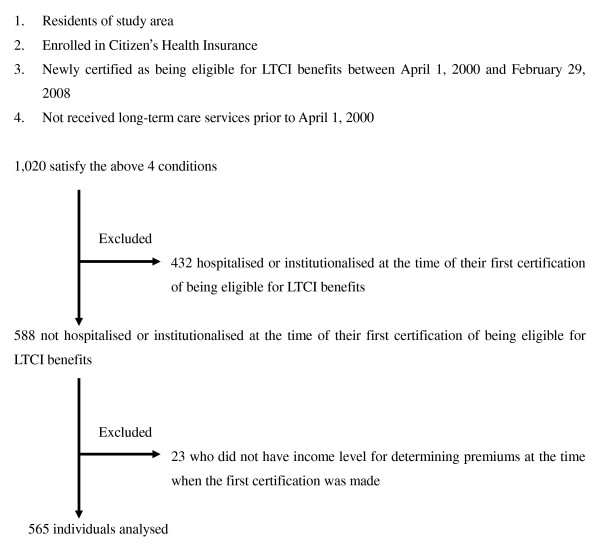
**Selection of analysed individuals**.

The subjects were drawn from a total of 1,020 individuals who were enrolled in the Citizen's Health Insurance in the study area, who had not been receiving long-term care services prior to April 1, 2000, and had been newly certified as eligible for LTCI benefits between April 1, 2000 (when LTCI was implemented), and February 29, 2008. Of the total, 432 who were hospitalised or institutionalised at the time of their first certification were excluded in order to standardise the base-line characteristics. The average eligibility level of those who were already hospitalised or institutionalised at the time of the first eligibility certification was 2.03 and significantly higher than 0.87 for those who were living in the community (the lowest eligibility level being 0, the highest 5). Twenty-three who did not have data for their income level were also excluded. The remaining 565 people were analysed. The sample had to be limited to those enrolled in the Citizen's Health Insurance program, which enrolls 80.5% of the population 70 and over, because record linkage with LTCI was only possible for this group.

### Preparation of data set

For all 1,020 subjects, information on long-term care services and medical services for each month from April 1, 2000 to February 29, 2008 was collected from the claims data of LTCI and Citizen's Health Insurance. The records for the two datasets were linked for each individual.

The information on long-term care services included sex, month and year of birth, start/end date of eligibility, eligibility level, date of death/removal, long-term care services code, month and year when long-term care services were provided, long-term care services claims amount, and LTCI premium amounts (based on income level).

The information on medical services was residential code (Town A or B); monthly medical care services claims amount for hospitalisation, outpatient care, dental care, dispensary, visiting nurse, and osteopathic therapy; and the dates of hospital admission and discharge.

### Measures

#### Outcome variable

The outcome variable was the number of months from the first certification of being eligible for LTCI benefits until the occurrence of an "event" or censoring. Events were hospitalisation, institutionalisation or death from the initial certification up until February 29, 2008. Death was included as an event because it is the worst adverse outcome and excluding deaths at home would bias the sample. In cases when there were no events by February 29, 2008, censoring was done at that time, or at the time the subject moved out of the area. "Hospitalisation" refers to being admitted to a hospital; "institutionalisation" to being admitted to a nursing home, health services facility for the elderly, or sanatorium-type medical care facility. Admission to group homes for the elderly with dementia and residential care facilities for the elderly requiring care, which administratively are defined as community-based care, were nearly all for the long-term, and were therefore included in "institutionalisation". Threshold expenditure amounts (i.e. 100,000 yen for the initial month, about $1,200 at the current exchange rate of US$1 = ¥82) were set for "hospitalisation" and "institutionalisation" in order to eliminate short episodes such as for examinations or treatment of mild trauma.

#### Predictor variables

The main predictor variables were the use of home and community-based services and of medical services. These variables were constructed from expenditure data in the following way.

Average monthly expenditures were calculated for outpatient medical expenditures, total home and community-based services expenditures, and expenditures for home-visit and day-care types of services, respectively. The cumulative amounts of outpatient medical expenditures, total home and community-based services expenditures, and expenditures for home-visit and day-care types of services for each analysed individual from certification until hospitalisation, institutionalisation, death or censoring were divided by the number of months and converted to the amount per month. "Outpatient medical expenditures" are the total for outpatient medical expenditures, dental expenditures, visiting nurse expenditures (when billed to health insurance), medication expenditures, and osteopathic therapy expenditures, calculated from their monthly claims amount. Total home and community-based services expenditures, and expenditures for home-visit and day-care types of services are calculated from the claims amount for the corresponding type of service. The detail of long-term care services included in all home and community-based services and home-visit and day-care types of services are shown in the tables and their legends.

Average monthly outpatient medical expenditures were divided into three levels: low, medium, and high. Non-users are included in the low expenditure group because Japanese tend to visit physicians for minor complaints [47]; thus their basic characteristics are likely to be substantively the same as non-users. Divisions into levels were calculated so that the number of subjects in each was equal. However, for LTCI services, non-users were grouped into a separate level because they may have a different rationale for undergoing the certification process if they subsequently had not used any services. The users group was divided equally into three levels of low, medium, and high expenditures.

Use or no use was recorded for each of the home and community-based services: home-help (including home-visit bathing), visiting nurse, management and guidance, day care, day rehabilitation, respite care and rental services for assistive devices. Services were considered to be used when they were billed and paid. In the study area, two services--visiting rehabilitation and small scale community-based multiple services centres--were not available.

#### Demographic variables

The demographic variables of sex, age, eligibility level for LTCI, area of residence, income level for determining LTCI premiums at time of the first certification of long-term care benefits, and year of the first certification were also taken as predictor variables. In this study, in order to maintain consistency in eligibility levels from April 1, 2000 to February 29, 2008, Support level 1 continued to be counted as Support level, but Support level 2 was grouped with Care level 1 after April 1, 2006, when the number of levels was increased from six to seven. These six levels were then combined into the three levels of Support level, Care levels 1 and 2, and Care levels 3 to 5 in order to have sufficient numbers for each level.

#### Statistical analysis

##### Main analysis

To calculate a hazard ratio for hospitalisation and institutionalisation using a Cox proportional hazard model, three analyses were carried out.

The outcome was the time to hospitalisation, institutionalisation or death from the time of the first certification of being eligible for LTCI benefits. In order to investigate the overall effect of home and community-based services (Analysis 1), the sex, age and eligibility level at time of the first certification of long-term care benefits were entered in a model as predictor variables. The other predictor variables were selected from the area of residence and income level at time of the first certification of long-term care benefits, the year of the first certification, outpatient medical expenditures and total home and community-based services expenditures by using a forward stepwise method. Variables were entered in a model if p < 0.05 and removed from a model if they became p > 0.10.

To observe the effects for each type of home and community-based service, a similar analysis (Analysis 2) was done with use or no use of each type of service replacing average monthly total home and community-based services expenditures in Analysis 1.

To observe the effects of home-visit and day-care types of services, respite care, and rental services for assistive devices, a similar analysis (Analysis 3) was made in which average monthly expenditures for home-visit and day-care types of services, use or no use of respite care and rental services for assistive devices were used as predictor variables, replacing average monthly total home and community-based services expenditures in Analysis 1.

##### Subgroup analysis

Analysis 2 was made with only individuals certified as needing long-term care of Support level or Care levels 1 or 2 (individuals certified as having light need for long-term care). This analysis was made because in some previous studies [20,22,24], observations of the effect of each home and community-based service on eligibility level were limited to individuals certified as having light need for long-term care.

The statistical software package SPSS Statistics Ver. 17.0 was used in the analysis.

### Ethical considerations

The data used in this study were provided by the insurer as linkable anonymous data from which personal information had been removed. This study was reviewed and approved by the ethics committee of the Keio University School of Medicine.

## Results

### Characteristics of subjects

The descriptive statistics of the outcome variable and predictor variables are shown in Tables 2 and 3. The mean age (years) ± standard deviation at the time of the first certification was 81.4 ± 6.7. The median was 82.0 (range 61.8-98.1). Among all the analysed individuals, 99.3% were ≥ 65 years old, and 91.9% were in Support level or Care level 1 or 2 (Table 2).

**Table 2:** Cohort characteristics (N = 565)

		N	%
**Sex**	Male	195	34.5
	Female	370	65.5

	<75	97	17.2
	<65	4	0.7
	65-70	31	5.5
Age*^1^	70-75	62	11.0
	75-80	125	22.1
	80-85	166	29.4
	85-90	126	22.3
	≧90	51	9.0

	Support level*^9^	247	43.7
Eligibility level*^1^	Care level 1·2*^10^	272	48.1
	Care level 3·4·5	46	8.1

Living area*^1^	Town A	383	67.8
	Town B	182	32.2

	Low*^11^	276	48.8
Income levels for determining premiums*^1^	Middle*^12^	210	37.2
	High*^13^	79	14.0

	2000	152	26.9
	2001	37	6.5
	2002	58	10.3
	2003	56	9.9
**Year of the first certification**	2004	54	9.6
	2005	56	9.9
	2006	75	13.3
	2007	65	11.5
	2008	12	2.1

	Low: 0-24,000	184	32.6
**Average monthly outpatient medical expenditures (yen)**	Middle: 24,000-53,000	189	33.5
	High: ≧53,000	192	34.0

	0	168	29.7
Average monthly total home and community-based services expenditures(yen)^*2^	Low: 189-18,000	132	23.4
	Middle: 18,000-36,000	131	23.2
	High: ≧36,000	134	23.7

	0	400	70.8
Average monthly home-visit type services expenditures(yen)*^3^	Low: 36-9,000	57	10.1
	Middle: 9,000-20,000	55	9.7
	High: ≧20,000	53	9.4

	0	290	51.3
Average monthly day-care type services expenditures(yen)*^4^	Low: 189-16,000	88	15.6
	Middle: 16,000-28,000	93	16.5
	High: ≧28,000	94	16.6

**Each home and community-based service use or not**			
	
Home-help*^5^	Use	145	25.7
Visiting nurse*^6^	Use	51	9.0
Management & guidance*^6^	Use	15	2.7
Day care*^7^	Use	230	40.7
Day rehabilitation*^6^	Use	66	11.7
Respite care*^8^	Use	108	19.1
Rental services for assistive devices*^6^	Use	87	15.4

**Outcome**			
	
	No event before 29 Feb.2008	155	27.4
**Censor**	Move out	4	0.7
	
	Subtotal	159	28.1
	
	Hospitalisation	327	57.9
**Event**	Institutionalisation	55	9.7
	Death	24	4.2
	
	Subtotal	406	71.9

*1 Data when the insured person is certified as being eligible for long-term care insurance benefits for the first time.

*2 Consists of expenditures of Home-help, Home-visit bathing, Visiting nurse, Management & guidance, Day care, Day rehabilitation, Respite care and Rental service for assistive devices.

*3 Consists of expenditures of Home-help, Home-visit bathing, Visiting nurse and Management & guidance.

*4 Consists of expenditures of Day care and Day rehabilitation.

*5 Includes Home-visit bathing. After 2006, "Preventive care" and "Night care" are included.

*6 After 2006, "Preventive care" is included.

*7 After 2006, "Preventive care" and "Services for the elderly with dementia" are included.

*8 Consists of regular and medically-related Respite care. After 2006, "Preventive care" is included.

*9 After 2006, Support level means Support level 1.

*10 After 2006, Care level 1 includes Support level 2.

*11 Both individual and household exempt from community tax.

*12 Individual exempt from community tax, household pays community tax.

*13 Individual pays community tax.

All years are fiscal years; April to March.

**Table 3:** Descriptive statistics of outcome and expenditure variables (N = 565)

	mean	mediam	standard devation	maximum	minimum
Observation period (months)*^1^	19.5	13.2	20.1	94.9	0.03
**Average monthly expenditures (yen)**					
**Outpatient medical services**	57,004	36,807	97,462	1,152,831	0
Total home and community-based services*^2^	25,560	15,657	34,637	255,009	0
Home-visit type services*^3^	8,002	0	23,682	210,097	0
Day-care type services*^4^	12,041	0	17,780	127,506	0

*1 Time to hospitalisation, institutionalisation, death or censoring from the first certification of being eligible for long-term care insurance benefits.

*2 Consists of Home-help, Home-visit bathing, Visiting nurse, Management & guidance, Day care, Day rehabilitation, Respite care and Rental services for assistive devices.

*3 Consists of Home-help, Home-visit bathing, Visiting nurse and Management & guidance.

*4 Consists of Day care and Day rehabilitation.

The average monthly outpatient medical expenditure per analysed individual was 57,004 yen, with a median value of 36,807 yen (Table 3). For the mid-level expenditure, the amount was 24,000-53,000 yen (Table 2). The national average for those in the medical care for the elderly program was about 22,000-23,000 yen in 2001 and 2007 (Table 1). Because the individuals analysed had been certified as being eligible for LTCI benefits, their average monthly outpatient medical expenditures were likely to be higher than that of the national level.

The average monthly total home and community-based services expenditure per analysed individual was 25,560 yen, with a median value of 15,657 yen (Table 3). The mid-level amount was 18,000-36,000 yen (Table 2) which was less than the national average per recipient of home and community-based services, which was between 47,000 and 49,000 yen in 2001 and 2007 (Table 1).

### Predictors of admission to hospital or long-term care institution or death

#### Main analysis (Table 4)

**Table 4:** Adjusted hazard ratios (HR) and 95% confidence interval (CI) for admission to hospital or long-term care institution or death (N = 565)

Analysis 1: The overall effect of home and community-based services				Analysis 2: The effects of each type of home and community-based service			
	**Adjusted HR**	**95% CI**	**p-value**		**Adjusted HR**	**95% CI**	**p-value**

**Sex**			0.007	**Sex**			0.006
Male	1			Male	1		
Female	0.749	0.607-0.924		Female	0.742	0.600-0.917	

Age*^1^			0.003	Age*^1^			0.001
< 75	1			< 75	1		
75 - 80	1.237	0.893-1.713	0.201	75-80	1.231	0.884-1.715	0.219
80 - 85	1.406	1.023-1.934	0.036	80 - 85	1.436	1.035-1.991	0.030
85 - 90	1.435	1.041-1.979	0.027	85-90	1.526	1.103-2.111	0.011
≧ 90	2.168	1.464-3.212	0.000	≧ 90	2.261	1.514-3.378	0.000

Eligibility level*^1^			0.014	Eligibility level*^1^			0.000
Support level*^2^	1			Support level*^2^	1		
Care level 1·2*^3^	1.177	0.946-1.465	0.143	Care level 1·2*^3^	1.314	1.057-1.634	0.014
Care level 3·4·5	1.769	1.199-2.611	0.004	Care level 3·4·5	2.154	1.450-3.199	0.000

Living area*^1^			0.047	Living area*^1^			0.045
Town A	1			Town A	1		
Town B	0.804	0.648-0.997		Town B	0.801	0.645-0.995	

**Year of the first certification**	0.946	0.902-0.992	0.022	**Year of the first certification**	0.942	0.900-0.986	0.011

Income level for determining premiums*^1^		Not Selected		Income level for determining premiums*^1^		Not Selected	

**Average monthly outpatient medical expenditures (yen)**			0.000	**Average monthly outpatient medical expenditures (yen)**			0.000
Low: 0 - 24,000	1			Low: 0 - 24,000	1		
Middle: 24,000 - 53,000	1.226	0.947-1.587	0.122	Middle: 24,000 - 53,000	1.191	0.920-1.542	0.185
High: ≧ 53,000	2.138	1.663-2.749	0.000	High: ≧ 53,000	2.149	1.668-2.769	0.000

Average monthly total home and community-based services expenditures*^4^	(yen)		0.005	Day care*^5^			0.088
0	1			Not use	1		
Low: 189 - 18,000	0.700	0.526-0.931	0.014	Use	0.821	0.655-1.029	
Middle: 18,000 - 36,000	0.587	0.436-0.791	0.000	Respite care*^6^			0.013
High: ≧36,000	0.722	0.542-0.960	0.025	Not use	1		
				
	Akaike' s Information Criteria(AIC) = 4434.401			Use	0.714	0.547-0.931	
				
				Rental services for assistive devices*^7^			0.011
				Not use	1		
				Use	0.703	0.537-0.921	
				
				Home-help*^8^		Not Selected	
				
				Visiting nurse*^7^		Not Selected	
				
				Management & guidance*^7^		Not Selected	
				
				Day rehabilitation*^7^		Not Selected	
				
					AIC = 4423.682		

Outcome variable was the number of months from the first certification of being eligible for long-term care insurance benefits until hospitalisation, institutionalisation, death or censoring in both Analysis 1 and 2. All years are fiscal years; April to March.

*1 Data when the insured individuals are certified being eligible for long-term care insurance benefits for the first time.

*2 After 2006, Support level means Support level 1.

*3 After 2006, Care level 1 includes Support level 2.

*4 Consists of expenditures of Home-help, Home-visit bathing, Visiting nurse, Management & guidance, Day care, Day rehabilitation, Respite care and Rental services for assistive devices.

*5 After 2006, "Preventive care" and "Services for the elderly with dementia" are included.

*6 Consists of regular and medically related Respite care. After 2006, "Preventive care" is included.

*7 After 2006, "Preventive care" is included.

*8 Includes Home-visit bathing service. After 2006, "Preventive care" and "Night care" are included.

The results of the analysis using a Cox proportional hazard model are shown in Table 4 (the results of Analysis 3 are essentially the same as those of Analysis 2, and so they are not shown).

In Analysis 1, there tended to be more hospitalisation or institutionalisation in men than in women, in those aged ≥ 80 years than <75 years, and in those with Care levels from 3 to 5 than Support level (HR: 1.77, 95% CI: 1.20-2.61). The residents of Town B were less likely to be hospitalised or institutionalised than the residents of Town A (HR: 0.80, 95% CI: 0.65-0.997). Those who were first certified later were less likely to be hospitalised or institutionalised (HR: 0.95, 95% CI: 0.90-0.99). The group with high average monthly outpatient medical expenditures was more likely to be hospitalised or institutionalised than the low expenditure group (HR: 2.14, 95% CI: 1.66-2.75). For average monthly total home and community-based services expenditures, the low expenditure (HR: 0.70, 95% CI: 0.53-0.93) and mid-level expenditure (HR: 0.59, 95% CI: 0.44-0.79) and high expenditure (HR: 0.72, 95%CI: 0.54-0.96) groups were all less likely to be hospitalised or institutionalised than the home and community-based services non-users group. No other predictor variables were selected.

In Analysis 2, the result was similar to that in Analysis 1 except for a greater tendency for hospitalisation or institutionalisation in Care level from 1 to 5 than in Support level. Hospitalisation and institutionalisation were less likely among users of respite care than non-users (HR: 0.71, 95% CI: 0.55-0.93), and among users of rental services for assistive devices than non-users (HR: 0.70, 95% CI: 0.54-0.92). Day care was selected, but was not statistically significant. No other predictor variables were selected.

The results of Analysis 3 were the same as those for Analysis 2.

#### Subgroup analysis (limited to individuals certified as having light need for long-term care)

For each home and community-based service, users of day care had fewer hospitalisations or institutionalisations than non-users (HR: 0.77, 95% CI: 0.61-0.98, p = 0.032). The results for other predictor variables were the same as in Analysis 2 of the main analysis.

## Discussion

We studied the impact of home and community-based services on hospitalisation and institutionalisation of individuals certified as being eligible for LTCI benefits for the first time following the introduction of public LTCI. The presence of a disease and its severity at the time of the first certification could be confounding factors, since they could lead to the hospitalisation or institutionalisation of the subject [11,15,48-58]. In our study, we took this factor into consideration by adjusting for outpatient medical expenditures, which would serve as an indicator of the severity of illness. The group with high average monthly outpatient medical expenditures was more likely than the low expenditure group to be hospitalised or institutionalised. This result and the results of demographic variables were reasonable.

Those who used home and community-based services, and respite care and rental services for assistive devices in particular, were less likely than non-users to be hospitalised or institutionalised. Thus, the policy objectives of LTCI appear to have been met especially for those using these services. Regarding assistive devices, Tajika et al. had reported that among individuals certified as having light need for long-term care, users of rental services for assistive devices tend to show a greater decline in eligibility level than non-users [20]. This observation was used to support the removal of rental services for wheel-chairs, motorised beds and other devices that might induce disuse syndrome from LTCI benefits for those in the lighter eligibility levels in 2007. However, our study shows that they had a beneficial effect, perhaps because the negative effect of some devices was less than the positive effect of using canes and walking aids which prevent decline. This indicates a need to differentiate among devices. For day care, when the subjects are limited to individuals certified as having light need for long-term care, a positive effect was seen, which supports the 2007 LTCI revision that promoted these services.

Two possible mechanisms may explain the effects of home and community-based services in preventing hospitalisation and institutionalisation. One is that home and community-based services prevent a decline in the physical and mental state of individuals certified as needing long-term care (prevention of decline) [14] and the other is that these services reduce the care burden of caregivers, allowing them to maintain their ability to provide care (maintenance of caregivers' ability) [26,59,60]. The effect of respite care could mainly be attributed to the latter [24,26,59-61], and not to the former [24,62]. Assistive devices, if used appropriately, should have the former effect [14,63]. The effect of day care among individuals certified as having light need for long-term care could be either maintenance of caregivers' ability [26,59,60,64,65], prevention of decline of physical and mental functions [14,22,65-68], or both.

## Limitations

The population studied was limited to those enrolled in the CHI, composing 80.5% of the total, and to those who had been certified as eligible for LTCI. Those who had not been certified were not included but this was unavoidable from the study design. Those who had moved outside of the two municipalities could not be followed-up, but they composed only 0.7% of the total. Among those in the sample, the data on service use should be complete because they were from the claims forms.

Average monthly expenditures for home and community-based services tended to increase somewhat as the time from the first certification elapsed, with the amount being ¥15,400 for the first three months and ¥21,800 for the last three months. This factor was not considered when making our analysis. However, since the difference between the two remained within the ranges of the three levels of expenditures and the hazard ratios for the three levels were almost the same (0.7), the magnitude of these changes are not likely to have impacted on our results.

Since this study was a retrospective cohort study, and not a randomised controlled trial, we could not verify any causal relationships. The possibility that the results were biased by residual and unmeasured confounding cannot be entirely ruled out.

The presence or absence of family members living with the individual who may provide support, and their caregiving ability, have been reported to have an impact on decline [69,70] and on hospitalisation and institutionalisation [26,52,60,71,72]. These factors could not be analysed in our study because the data were not available, but they should be included in future studies.

Our study was conducted in farming communities in one region of Japan and it should be replicated in urban communities and other regions.

## Conclusions

In this study, the impact of home and community-based services on hospitalisation and institutionalisation of individuals certified as being eligible for LTCI benefits for the first time was analysed after adjusting for demographic variables and outpatient medical expenditures. The results showed that users of home and community-based services were less likely than non-users to be hospitalised or institutionalised. Among the types of home and community-based services, users of respite care and rental services for assistive devices were less likely to be hospitalised or institutionalised than non-users. When subjects were limited to individuals certified as having light need for long-term care, hospitalisation and institutionalisation were also less likely for users of day care than for non-users. Therefore, respite care, rental services for assistive devices and day care were effective in preventing hospitalisation and institutionalisation. Our results suggest home and community-based services contribute to the goal of the LTCI system of encouraging individuals certified as being eligible for LTCI benefits to live independently at home for as long as possible.

## Abbreviations

LTCI: long-term care insurance; HR: hazard ratio; CI: confidence interval; CHI: Citizen's Health Insurance.

## Competing interests

The authors declare that they have no competing interests.

## Authors' contributions

NT, KY and NI developed the original idea for this study. NT extracted the data, performed the statistical analysis and wrote the original manuscript. KY and NI provided supervision during the entire process. All authors have read and approved final manuscript.

## Pre-publication history

The pre-publication history for this paper can be accessed here:

http://www.biomedcentral.com/1472-6963/10/345/prepub
